# Channel crossing: how are proteins shipped across the bacterial plasma membrane?

**DOI:** 10.1098/rstb.2015.0025

**Published:** 2015-10-05

**Authors:** Ian Collinson, Robin A. Corey, William J. Allen

**Affiliations:** School of Biochemistry, University of Bristol, University Walk, Bristol BS8 1TD, UK

**Keywords:** SecYEG, Tat, protein translocation, protein secretion

## Abstract

The structure of the first protein-conducting channel was determined more than a decade ago. Today, we are still puzzled by the outstanding problem of protein translocation—the dynamic mechanism underlying the consignment of proteins across and into membranes. This review is an attempt to summarize and understand the energy transducing capabilities of protein-translocating machines, with emphasis on bacterial systems: how polypeptides make headway against the lipid bilayer and how the process is coupled to the free energy associated with ATP hydrolysis and the transmembrane protein motive force. In order to explore how cargo is driven across the membrane, the known structures of the protein-translocation machines are set out against the background of the historic literature, and in the light of experiments conducted in their wake. The paper will focus on the bacterial general secretory (Sec) pathway (SecY-complex), and its eukaryotic counterpart (Sec61-complex), which ferry proteins across the membrane in an unfolded state, as well as the unrelated Tat system that assembles bespoke channels for the export of folded proteins.

## 1. Background

Energy conservation through chemiosmosis and oxidative phosphorylation provides the means to control the chemistry and dynamics of the cell. The free energy available from ATP hydrolysis and electrochemical gradients is coupled to the transport of biochemical metabolites and polymers across membranes according to the specific requirements of the cell: for import, export or between internal compartments in eukaryotic cells. Proteins, for instance, often need to be transported following (or during) their synthesis in the cytosol across and/or into biological membranes for secretion, membrane insertion and import into organelles such as chloroplasts and mitochondria. In every living cell, protein secretion and membrane protein insertion occurs via the general protein secretion, or Sec, machinery. This is the case at the plasma membranes of bacteria and archaea, the chloroplast thylakoid membrane and the endoplasmic reticulum (ER) of eukaryotes.

Between one-quarter to a third of all proteins either cross or insert into biological membranes [1,2]; most of these use the Sec protein machinery, which transports proteins in an unfolded state. The Sec protein-conducting channel is formed by a hetero-trimeric membrane protein complex: the SecY-complex in bacteria, archaea and plant plastids, and Sec61 in eukaryotes. The channel acts either co-translationally, engaging translating ribosomes and forming a ribosome nascent chain complex (RNC), or post-translationally, which involves the binding of specialized energy-transducing factors. The co-translational system is the primary pathway for protein secretion in eukaryokes [3–5], and for membrane protein insertion in all domains of life [6]. The post-translational pathway, meanwhile, is used mainly by prokaryotes for protein secretion [7], although an increasing number of eukaryotic proteins have also been shown to follow this pathway [8,9].

**Figure 1.**
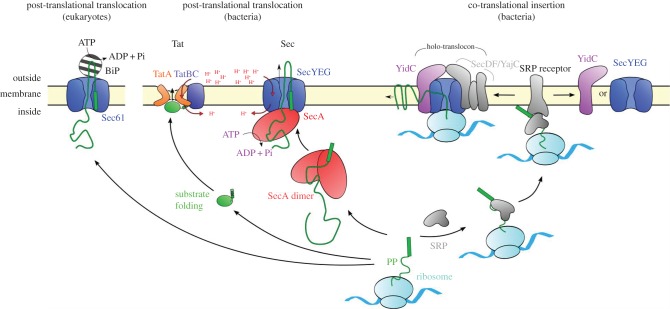
Pathways for protein transport. From left to right: BiP-mediated post-translational translocation in eukaryotes; post-translational translocation of folded (Tat system) and unfolded (Sec system) proteins in bacteria; co-translational insertion in bacteria through the HTL complex or its individual components. Note that SecYEG has been shown here as a monomer for clarity.

Both types of secretion are initiated when the N-terminal region of a pre-protein is targeted to the SecY/Sec61 complex at the plasma (prokaryotes) or ER (eukaryotes) membrane (figure 1). The central motif by which substrates are recognized is a stretch of hydrophobic amino acids preceded by one or more positively charged residues [10]: for nascent membrane proteins, the hydrophobic region is usually the first transmembrane helix (TMH)—the signal anchor (SA), while secreted substrates possess an N-terminal signal sequence (SS) which is cleaved by signal peptidase following translocation. These signals are discriminated mainly on the basis of their hydrophobicity. In bacteria, the more hydrophobic nascent SAs [11], especially those with more positively charged N-termini [12], are recognized by the signal recognition particle and directed to their cognate receptor for co-translational insertion, whereas pre-secretory proteins engage with the SecA motor ATPase, either directly or *via* chaperones (e.g. SecB), for post-translational transport [11].

The membrane protein insertion process involves the passage of TMHs directly from the ribosomal exit tunnel, through the translocon and laterally into the lipid bilayer (figure 1). This process is facilitated by ancillary components which associate with the core complex to aid debarkation into the membrane: the Sec61-complex cooperates with the transmembrane protein associated membrane protein (TRAM) [5,13], while the bacterial SecYEG core complex associates with SecDF–YajC and the membrane protein ‘insertase’ YidC to form the holo-translocon (HTL) [14–17] capable of both protein secretion and membrane protein insertion [18]. Furthermore, SecA has been implicated in the export of some regions of membrane proteins, such as large periplasmic loops [19,20]. The membrane protein insertion process *per se* has been reviewed recently [21]; this paper is primarily concerned with the post-translational secretion pathway, best described for the bacterial system.

In bacteria, secretory proteins are targeted to the translocation machinery of the cytosolic membrane. Translocation through the SecY-complex occurs following the activation of the channel by the SS (see §2*c*) [22,23] and requires the pre-proteins to be in an unfolded conformation [24]. Secretory proteins that are required to fold in the cytosol—usually due to the incorporation of cofactors—are transported across the membrane by a separate general export pathway: the twin arginine translocation, or Tat machinery (figure 1) [25]. Just like the classical secretory (Sec) proteins, the Tat substrates also have an N-terminal cleavable SS, distinguished by a twin arginine motif at the N-terminus [26]. Recent breakthroughs in structural determination of the Tat components provide hope that the molecular mechanism underlying this process may now also be addressed [27,28].

## 2. Structure and function of the bacterial SecY translocon

### (a) Structure of SecYEG

**Figure 2.**
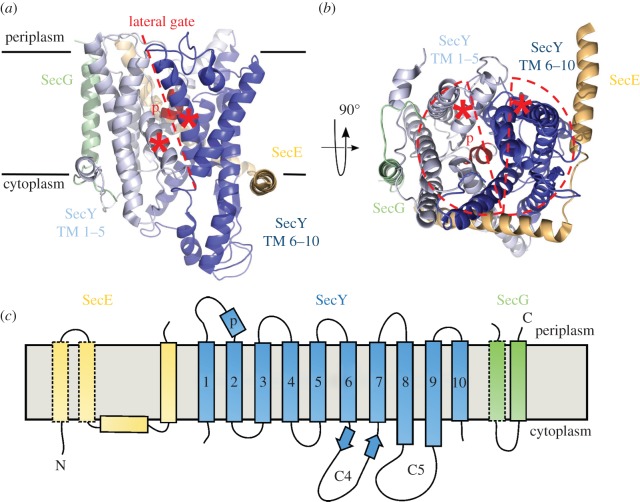
Structure of SecYEβ complex from *M. jannaschii*. (*a*) SecYEβ viewed from the side, in position in the lipid bilayer (black lines). TMHs 1–5 of SecY are coloured light blue, TMHs 6–10 dark blue, with the plug helix (labelled ‘p’) in red, SecE in wheat colour and SecG/β in green. The LG is indicated with a dashed red line, and the lateral gate (LG) helices are marked with asterisks. Structural data from [29]. (*b*) As in panel (*a*) but viewed from the cytoplasm. Red semicircles have been superimposed to indicate the separate halves of SecY. (*c*) Schematic of *E. coli* SecYEG. SecE is in yellow, SecY in blue with the TMHs numbered and the primary cytoplasmic loops (C4 and C5) and plug (p) marked, and SecG is green. Conserved regions are shown in solid lines and the non-conserved in dashed lines.

The first high-resolution structure of the Sec translocon was of the *Methanococcus jannaschii* complex (SecYEβ; figure 2*a*,*b*: PDB code 1RHZ) [29]. Although solved in the absence of any translocation partners, this structure provided many clues as to how the translocon functions: how the SS docks, how the complex opens to allow the passage of polypeptides through, and ferries TMHs laterally into the membrane, all while preserving essential membrane ion gradients.

**Figure 3.**
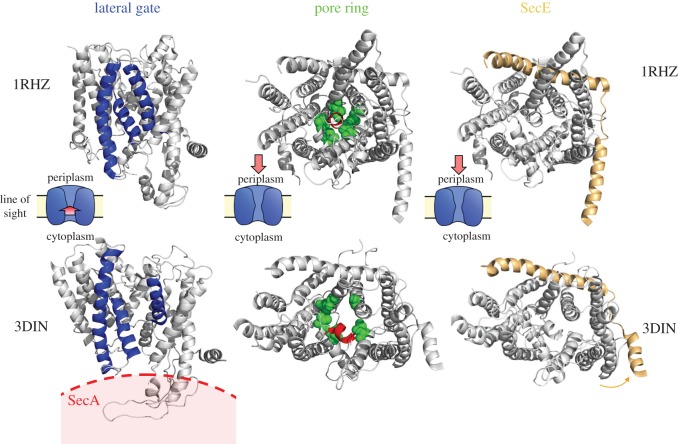
Comparison of resting and activated SecYEG. Left: Structures comparing the SecY LG in both resting (1RHZ [29]) and activated (3DIN [31]) states, as viewed from the side. SecYEG is coloured grey apart from the three LG helices, which are blue. The activated SecYEG structure (below) has a much wider LG than that of the resting structure (above). Note that SecA in 3DIN has been omitted for clarity; its position is marked in red. Middle: As in the left panels, but viewed from the periplasm, with the pore ring residues coloured as green spheres and the plug as red cartoon. Right: As before but with all of SecY and SecG in grey and SecE shown in wheat colour highlighting the mobility of the SecE amphipathic helix.

**Figure 4.**
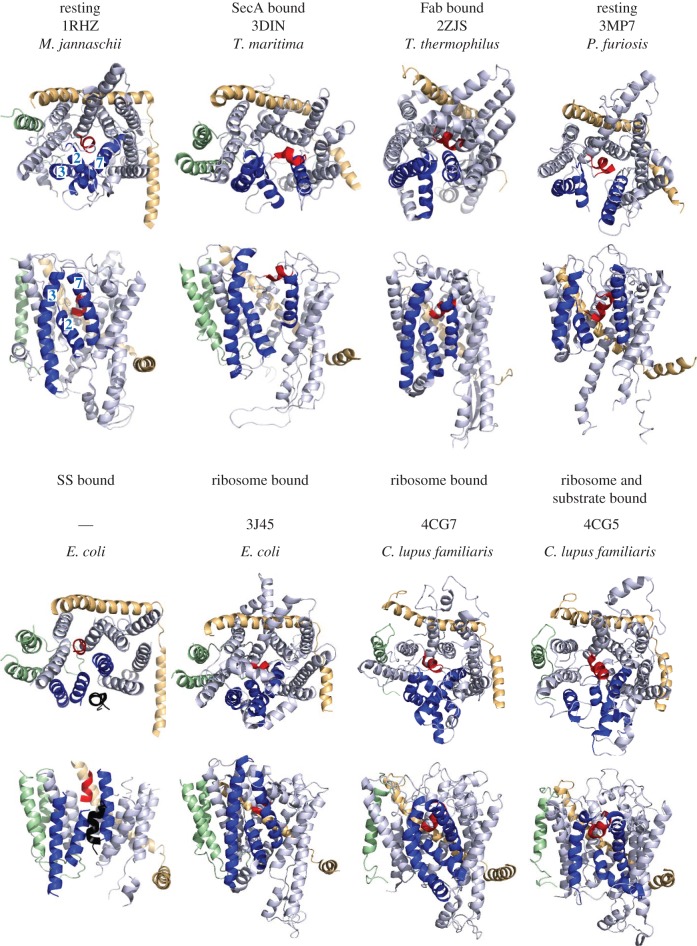
Comparison of SecYEG/Sec61 structures from various different studies. In each structure, SecY has been coloured in light blue, SecE in wheat and SecG in green, with the plug helix of SecY in red and the three SecY LG helices in dark blue (TMHs 2, 3 and 7; numbered accordingly in top left-hand panel). Where present, substrate helices are coloured in black (note that the substrate is not visible 4CG5, as the density was not assigned in the original structure).

SecYEG is a hetereotrimer, of which SecY is the largest subunit, forming the core of the transmembrane channel. SecY has 10 TMHs arranged in a pseudo-symmetrical crab-claw-like structure, connected by a hinge region between TMHs 5 and 6 [29] (figure 2*b*). Protein translocation occurs through an hourglass-shaped pore [30], constricted at the centre of SecY by the ‘plug’ (red helix in figure 2) and a ring of six inward facing hydrophobic residues (green spheres in figure 3) [29]. Together, they maintain the channel in a closed state in the absence of translocating polypeptide. The ‘plug’ is highly flexible; structural [29,31,32] and biochemical [33–35] evidence shows that it can sit either in the centre or at the periphery of the channel (figure 4). Presumably, the plug must move away from the centre of the channel during translocation (see §2*b*).

On the opposite side of the hinge connecting the two halves of SecY, there is a lateral gate (LG), between TMH 2 and 7/8. Separation of the two domains of SecY would widen the channel through the membrane as well as a gateway into the bilayer, necessary to allow the passage of proteins through and into the membrane (figure 2*b*; red asterisks mark approximate position of LG). SecE might help shore up the two SecY domains close to one another to maintain the channel and LG in a closed state [29]. SecE has between one and three TMHs, of which the tilted C-terminal is most conserved (figure 2*c*; beige TM, solid outline). Additionally, it contains an amphipathic helix that lies horizontally on the cytoplasmic face of the membrane and contacts the C-terminal ‘crab-claw’ of SecY (TMHs 6, 8 and 9) [29]. SecG (or Sec-β) has either one or two TMHs at the periphery of the complex and is required for efficient translocation [14,36].

### (b) Activation of SecYEG

**Figure 5.**
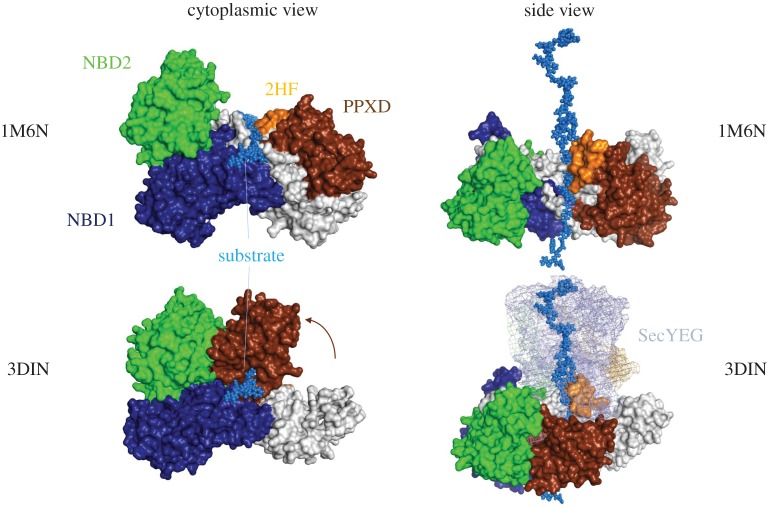
Structures of SecA in a resting state (above, 1M6N [43]) and bound to SecYEG (below, 3DIN [31]), viewed from the cytoplasm (left) and the side (right). Key domains (NBD1, NBD2, PPXD and 2HF) are coloured and labelled; white regions comprise other domains not mentioned in the text. A pre-protein substrate has been modelled into SecA using known cross-linking sites [44] and energy minimized (marine spheres); note that closing of the PPXD (brown arrow) forms a clamp around the substrate. In the SecYEG-bound structures, SecYEG is shown as a mesh (although it is obscured by SecA in the left panel), with the 2HF of SecA inserted into the channel.

In bacteria, SecYEG associates with the motor ATPase SecA during post-translational protein translocation [37]. SecA contains two nucleotide-binding domains (NBDs) with Walker A and B motifs [38,39], together forming the nucleotide-binding site (NBS), wherein ATP hydrolysis occurs [40], stimulated by SecYEG, acidic lipids and pre-protein substrates [41,42] (figure 5). Additional domains, thought to be important for transport, include the polypeptide cross-linking domain (PPXD) and two helix finger (2HF) [31,43] (figure 5), both of which contact the translocating polypeptide [44]. Based on the biochemical cross-linking sites [44], we have modelled and energy minimized a probable course for the peptide through SecA (blue spheres in figure 5).

A flagship structure in the field—*Thermotoga maritima* SecYEG bound to SecA, in complex with an analogue of ATP (ADP-BeF*_x_*)—revealed large conformational changes in both the motor and channel components [31] (PDB code 3DIN). The most striking effect of the interaction is the relocation of the PPXD of SecA towards NBD2 by about 25 Å (see figure 5, lower panels) and the insertion of the 2HF into the entrance of the SecY channel. The relocation of the PPXD activates the ATPase activity of the adjacent NBS [45] and forms a clamp for the translocating pre-protein [31]. For SecY, the most substantial consequence of this association is the partial opening of the channel and the LG (figure 3; left panels). These movements have been replicated in MD simulations [46] and shown to respond to the hydrophobicity of translocating substrates both *in silico* [47] and *in vitro* [48]. Recent electron cryo-microscopy (EM) studies of the ribosome-engaged Sec translocon show that the occupancy of a nascent chain does not necessarily bring about channel or LG opening (figure 4). This demonstrates that the LG is highly dynamic and responsive to the interacting partner and the translocation substrate.

The opening of the LG corresponds to a widening of the central pore, due to retraction of three of the six hydrophobic pore ring residues, located on TMHs 2 and 7 (figure 3; central panels, green spheres). This opening would permit the passage of unfolded polypeptide through the channel, while still forming a seal around the substrate to prevent the flow of small molecules and ions [49]. This SecA-induced widening of the pore ring, in turn, appears to destabilize the plug, which is seen to shift away from its central position in channel towards the periplasmic end of TMH 7 (figure 3; central panels, red helix) [31]. The association of the SS at the LG also brings about the displacement of the plug from its central blocking position [23].

Concerning the cytoplasmic loops of SecY (figure 2*c*), their prominence on the cytoplasmic face makes them important activation points for post- and co-translational translocation partners. SecA and ribosome binding results in substantial reordering of the largest and functionally most important loops, C4 and C5 [31,50]. The cytosolic amphipathic helix of SecE is also prone to perturbation upon SecA binding [31] (figure 3; right-hand panels). This tilting towards the periplasm may loosen the stabilizing grip of SecE around SecY and, thereby, permit the opening of the channel.

### (c) Unlocking of the complex

The SS is an allosteric activator of the SecY channel [22], acting as a ‘key’ to bring about a translocation-competent ‘unlocked’ state of the channel. A structure of membrane-bound SecYEG unlocked by a SS [23] shows that the most considerable change to the channel occurs in the normally tilted TMH 7, which straightens 40° towards the centre of the channel and bringing its outer (periplasmic) end 15 Å closer to TMH 10 (‘SS bound’ in figure 4). This tilting is distinct from the conformational changes induced by SecA and by the RNC (3DIN, 3J45, 4CG7 and 4CG5 in figure 4) [31,50,51]. However, the different structures are all marked by bending or displacement of TMH 7, suggesting that it may act as a ‘rudder’ responsible for channel activation, under the cooperative control of the N-terminal SS, and SecA or ribosomes via loops C4 and C5. For instance, the combined effects of the SS and SecA, relayed through TMH 7, could bring about the necessary displacement of the plug and opening of the channel prior to protein intercalation and transport. A set of protein localization (*prl*) mutations of SecYEG, initially identified by their ability to export pre-proteins with a defective SS, are thought to favour an activated state of the channel. The most potent of these, PrlA4 (a double mutation on TMHs 7 and 10 [52–54]), presumably does so through perturbation of the ‘rudder’, promoting its movement towards TMH 10 and thus the stabilization of the unlocked state.

### (d) Oligomeric state of SecYEG

For many years, opinions on the active oligomeric state of SecYEG have been divided. Various studies show that SecYEG dimers are stabilized by lipids and the environment of the membrane [55–57] and dimers interact more productively with SecA [58–60]. However, translocation proceeds through one copy of the pair [29,30,61], and the second redundant copy is dispensable [18,62]. Most likely, the interaction network of SecYEG is much more dynamic than structural studies can do justice: the SecYEG dimer exists, but is just one of multiple possible configurations that occur *in vivo*.

## 3. Mechanism and energetics of translocation

### (a) Steps on the protein export pathway

The secretion process *per se* can be divided into two distinct steps: initiation and translocation. The initiation step, presumably analogous to unlocking (see §2*c*), is crucial for the selectivity of the Sec machinery towards secretion substrates [3]. The SS is thought to act at the interface between the translocon and the lipid bilayer [63–65], more precisely at the LG [23]. For some, but not all proteins, amino-terminal fusion of a synthetic SS is sufficient for export through the Sec complex [66,67]. Conversely, provision of a synthetic SS *in trans* [22] or activating (*prl*) mutations (see §2*c*) can to some extent bypass the requirement for a SS. By contrast, the subsequent processive translocation of the remaining polypeptide, must by necessity be relatively non-specific: a wide variety of proteins pass through the Sec translocon, which may contain alternating stretches of bulky, hydrophobic and charged amino acids.

These two processes—initiation and translocation—are distinct in other ways as well. In bacteria, where both steps are mediated by SecA, non-hydrolysable analogues of ATP are sufficient for insertion of the signal peptide into the membrane [68], but ATP turnover is required for any subsequent translocation steps. The membrane proton motive force (PMF), meanwhile, appears to act exclusively at the later stages of translocation and can drive the translocation of trapped translocation intermediates even in the absence of ATP [68], although it may also have a role in orienting the SS [69].

An important question for understanding the overall secretion process in the cell is whether initiation or translocation is rate limiting: the latter case, for example, would need a way to recognize and rescue stalled intermediates. Kinetic experiments on the rate of translocation are key to answering this question; however, results have been contradictory. The translocation time has been determined to be proportional to the length of the substrate (approx. 270 amino acids per minute *in vitro*), with an initial lag phase also dependent on substrate length [70]. The presence of a lag phase is clear evidence that transport of the pre-protein through the channel is rate limiting for translocation: if initiation were the slowest step, no such lag would be observed. By contrast, an independent study found no evidence of a lag phase and suggested instead a single rate-limiting step for the entire translocation process [71]. These results can best be reconciled by the fact that PMF was absent in the former experiments, but present for the latter. As PMF profoundly stimulates the later stages of translocation [68], it could well be that initiation is rate limiting only in its presence.

A recent kinetic analysis followed up these results using substrates where processive translocation was slowed by stretches of poly-lysine [72]. Surprisingly, even this did not give rise to a lag phase, despite the overall rates of translocation slowing down. To explain this, the authors proposed a model where initiation is rate limiting, but initiated complexes are either rapidly translocated or rapidly released (i.e. initiation is slower than translocation and release) [72]. In the context of the cell, this hypothesis strikes us as very plausible: it eliminates the problem of blocked SecYEG translocons, which would be highly deleterious to cellular function and viability [73]. Furthermore, it is perfectly compatible with *in vitro* experiments: if the translocation or release steps are compromised—e.g. where PMF or other cellular components are missing—protein transport could become rate limiting for the overall process [70]. Indeed, given that the ATP consumption is around five ATP per amino acid for *in vitro* experiments in the absence of PMF [70]—much higher than any model for transport predicts (see §3*b*), and indeed higher than the cost of synthesizing the protein in the first place [74]—the experimental system is probably not accurately recapitulating the cellular process.

### (b) ATP-driven protein export: power stroke or diffusional ratchet?

The driving force for protein secretion is perhaps better understood in eukaryotic systems than in bacteria (for a recent, more detailed review, see [75]). In yeast, once translocation is initiated, the substrate pre-protein can diffuse backwards or forwards through the Sec61 channel through random Brownian motion. However, as the polypeptide emerges into the ER lumen, it is recognized and bound by the Hsp70 homologue BiP (figure 1) [76]. The binding of a bulky chaperone prevents retrograde diffusion of the pre-protein through the channel—backsliding—and ensures that the net direction of translocation is forward. Such a mechanism, which functions by vectorializing thermal energy, can be referred to as a Brownian motor (or Brownian ratchet). ATP hydrolysis in BiP subsequently allows dissociation and folding or downstream processing of the translocation substrate.

**Figure 6.**
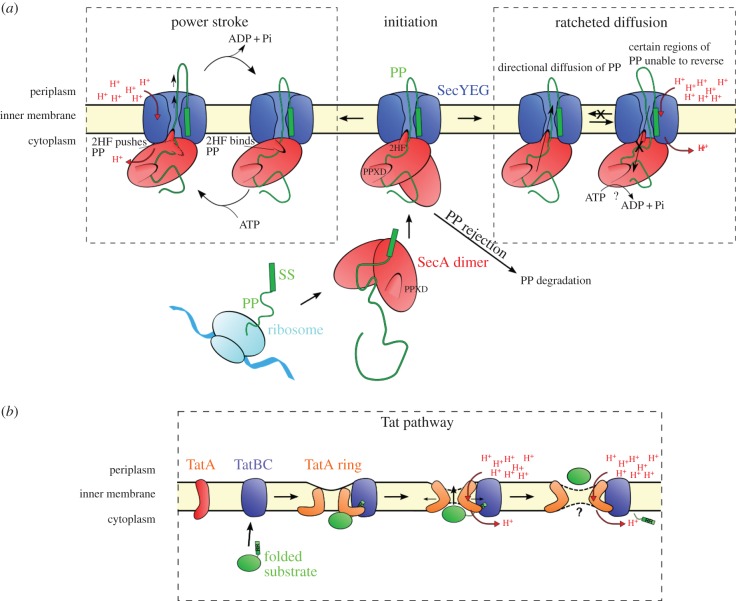
Proposed mechanisms of (*a*) Sec- and (*b*) Tat-mediated protein translocation. See §§3 and 4 for details.

Bacteria, in contrast, do not have the luxury of ATP on the distal side of the membrane; they must instead drive secretion from within the cytoplasm. Despite a plethora of proposed models for how SecA-driven translocation is accomplished (see §3*c*,*d*), the absence of direct experimental evidence has prevented any from gaining traction. At their core, these various models can be broken down into two types: power-stroke models—in which sequential steps in the ATPase cycle of SecA lead to binding, forward movement and release of the polypeptide chain (figure 6*a*, left)—and diffusion-based models, where turnover of ATP acts as a ratchet to bias the direction of pre-protein diffusion through the channel (figure 6*a*, right). The former mechanism is tempting in that it mimics the activity of DEAD-box helicases, to which the ATPase domains of SecA are related [43], although it should be borne in mind that the Sec machinery has no repeating phosphate backbone to cling on to. A ratcheting mechanism, meanwhile, would be more analogous to the proposed Sec61 pathway (above) [75]; however, in the absence of BiP or periplasmic ATP, it would need some other, as of yet unidentified way to prevent backsliding.

### (c) Power-stroke models of secretion

Translocation intermediates observed, many years ago, with low concentrations of ATP [68,77], were thought to be symptomatic of discrete step sizes of transport corresponding to a single turnover of ATP. This concept involving a step size of approximately 5 kDa (2–2.5 kDa for ATP binding and 2–2.5 kDa for hydrolysis [78]) persists. In practice, however, it has proved difficult to reconcile such a large step size—roughly 16 nm of linear (unfolded) peptide (assuming 0.11 kDa and 0.36 nm per amino acid)—with plausible conformational changes in SecA. The PPXD—the only domain of SecA shown to move far enough for such a large step size—can be immobilized without inhibiting translocation [45]. Most other models (see §3*d*) use some element of diffusion, and as such would not to give rise to such defined bands by SDS-PAGE, i.e. the intermediates would be of variable rather than fixed length.

It therefore seems most likely that the intermediate translocation products—only seen for some substrates and when ATP is limiting—represent local energy minima on the translocation pathway. Presumably, given the non-uniform nature of protein chains, some regions of substrate, e.g. those that are strongly hydrophobic [79] or positively charged [72,80], will be more difficult to translocate than others. Under conditions where translocation is compromised (i.e. low ATP, no PMF), these could well become stuck and give rise to defined bands following gel electrophoresis. Indeed, it was shown nearly 20 years ago that the pattern of bands can be altered by changing the substrate sequence [79]—contrary to the idea of a step size based purely on the mechanism of translocation.

The other central pillar of the power-stroke model was the observation that a large fragment of SecA becomes protease resistant when bound to SecYEG and pre-protein, but only in the presence of ATP or AMPPNP, not ADP [81]. This was interpreted as insertion and de-insertion of a 30 kDa part of SecA into the channel, with ATP binding pushing substrate into the channel, and hydrolysis causing its retraction. The advent of a structure of SecYEG–SecA complex [31] provided a perfect candidate for this 30 kDa domain: the 2HF is inserted deep into the channel opening, makes extensive contacts with the translocating pre-protein, and is critical for coupling ATP hydrolysis to directional movement [82,83]. However, given the range of different sequences that must be translocated, it is not clear how a single binding site could recognize them all. Furthermore, the tip of the 2HF can be cross-linked at the channel entrance at the centre of SecY without preventing translocation [84].

An alternative, plausible explanation for the observed protease resistance [81] is the tightness of the SecYEG–SecA interaction. SecA is known to bind with a higher affinity in the presence of AMPPNP than ADP [60], with a much slower off-rate [83]. Access to protease could therefore simply be a kinetic effect: tightly bound SecA does not spend enough time free in solution to be degraded. The effect of nucleotide state on the affinity of SecA for SecYEG could be evidence of a translocation mechanism driven by binding and release of SecA. However, based on the rate of release of SecA (approx. 0.03 s^−1^; [83]) relative to the rate of ATP turnover during translocation (7.6 s^−1^ [42]), it seems highly implausible that multiple rounds of SecA binding and release could be the main driver of translocation.

Another type of power-stroke model proposes that translocation might be driven by quaternary interactions between multiple copies of SecA [85–87]. The oligomeric state of SecA has been controversial for many years: while it is clearly a dimer in solution, many different dimer interfaces have been observed, and studies of its oligomeric state in the presence of other translocation components have produced conflicting results (for recent discussion, see [85,88,89]).

Recently, a thorough investigation of SecA dimerization during translocation concluded that interchange between various dimer states is required for the early stages of translocation—presumably initiation—but that subsequent monomerization is necessary for transit of the rest of the pre-protein [85]. The complexity of this model—invoking multiple different forms of SecA that interconvert—might explain the tangle of confusing and contradictory results that beset this topic: depending on experimental conditions and technique, different facets of SecA are brought to the fore. Further analysis of SecA oligomerization at different stages of the translocation cycle undoubtedly will be required to properly resolve this issue.

### (d) Diffusional models of secretion

Diffusional models of protein translocation have, in theory, many advantages over power-stroke models [90]. For one, they are potentially much faster—thermal motion is extremely rapid on the scale of proteins at physiologically relevant temperatures [90]. In addition, if the pre-protein is freely diffusing in one dimension through the channel, it should have much less requirement for sequence specificity—provided the channel is wide enough and does not interact too strongly with the substrate. And furthermore, it is easy to envisage how the PMF could cooperate to aid diffusion across the membrane, but much harder to see how it could affect a motor where ATP turnover causes transit of a fixed portion of substrate.

When evaluating the various mechanisms for translocation, it is useful to take a step back and consider the overall secretion requirements of a cell. A typical *E. coli* cell contains about 3 million proteins [91]. To achieve a doubling time of 20 min, and given that approximately 20% of the protein mass of an *E. coli* cell is located in the cell envelope [92]—mostly transported via SecYEG—this means export of approximately 30 000 proteins per minute. Combinatorial proteomics-based approaches to protein quantification gives a value for SecY that corresponds to about 500 copies per cell (187 ppm [93], using a *M*_w_ of 48 kDa, and on the assumption that an *E. coli* cell has a volume of 1 fl and contains 0.2 g ml^−1^ protein [91]); this is in remarkable agreement with early estimates [94]. Thus, roughly one pre-protein must be exported per translocon per second. Even given the rudimentary nature of this calculation, each translocon is probably exporting many pre-proteins per minute, which is difficult to reconcile with a stepwise mechanism coupled to ATP hydrolysis, on the basis that it would be far too low. Such speed seems far more in keeping with a diffusion-based model of translocation, particularly if the rate-limiting step for the entire process is initiation.

Historically, the step size artefact described above has been the main grounds for rejecting diffusional-based models [87]. The linear relationship between protein length and translocation time has also been taken as evidence for a fixed step length [70]; however, in practice any mechanism would, under conditions where transit though the pore is rate limiting, produce the same kinetic profile. Given the speed at which translocation must take place within the cell, we therefore believe that a diffusional model of protein secretion is most consistent with current evidence. Indeed, it has been shown already that some types of sequence diffuse freely through the SecY channel [83]—and diffusion is known to occur through the related Sec61-complex.

A major gap in the above logic is a structural rationale for how backsliding is prevented. Factors such as periplasmic chaperones or folding do not seem to be required for translocation; indeed, chaperones known to interact with translocation substrates post-secretion—such as SurA and Skp, which ferry proteins to the outer membrane—do not use ATP to drive their subsequent release (as there is no ATP in the periplasm, and no identified structural coupling to the cytosol). They must therefore use all their binding energy for downstream release of the substrate [95]. One clue lies perhaps in the clamp within SecA, formed by the PPXD and verified by cross-linking studies [44]. Although it can be cross-linked shut, preventing its involvement in a power stroke [45], the clamp could nonetheless open and close enough to influence the rate of diffusion. Perhaps this clamp is able to detect backsliding and close in response, using the energy of the ATPase cycle.

### (e) Stimulation of secretion by the proton motive force

SecA and its ATP turnover cycle have been the subject of countless papers. By contrast, the role of the PMF in secretion has been relatively overlooked—despite being demonstrated 25 years ago [37]. One reason for this is probably technical: it is much easier to explore ATP-dependent reactions, and impossible to produce a crystal structure with a PMF present. Another key factor is that while ATP turnover is absolutely critical to get translocation started, the PMF can be omitted. Nonetheless, to achieve the physiological rates of translocation that a cell needs (see §3*d*), PMF is clearly necessary.

Part of the effect of PMF on secretion is mediated by SecDF [96,97]. While the details are not clear, a structure of SecDF has been solved, revealing a large, mobile periplasmic domain. Furthermore, proton flow through the SecDF complex has been directly observed [97]. Taken together, these results led to the proposal of a model whereby proton flux gives rise to conformational changes in the periplasmic domains of SecD and SecF, which in turn pull or ratchet the substrate through the channel from the periplasmic side [97]. Such a model, while speculative, makes sense by analogy to BiP and could plausibly assist ATP-mediated translocation without being absolutely required.

SecDF is not the whole story though: PMF is still able to stimulate translocation even in reconstituted systems where SecDF is absent. Evidence suggests that both components of the PMF—the pH gradient (ΔpH, acidic outside) and a charge gradient (Δ*ψ*, positive outside)—are involved in translocation [98,99]. The mechanism, however, remains enigmatic: clearly, while Δ*ψ* could potentially assist passage of negatively charged substrates through the electrophoretic effect, it would equally hinder positively charges stretches. With regards to ΔpH, no convincing path for protons though SecYEG has yet been identified.

It is interesting to note that *prl* mutations of SecY, while stimulating overall translocation rate, are not further stimulated by PMF [100]. This has been taken to suggest that PMF is involved in activating the Sec machinery: if the machinery is already activated by mutation, then it has no further effect [100]. However, it would be surprising if the sole effect of PMF were a conformational change, given that it is a permanent feature of such an energy-transducing membrane.

## 4. Mechanism of the export of folded proteins through the Tat system

**Figure 7.**
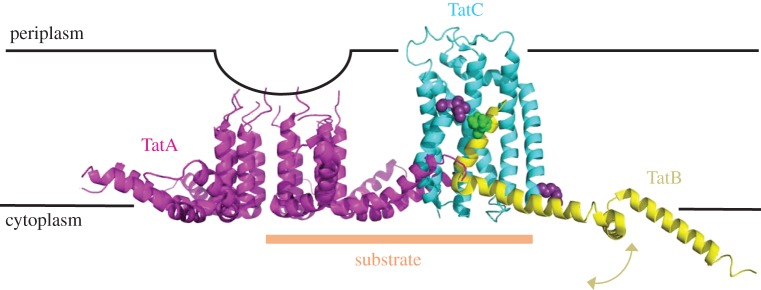
One possible arrangement of TatA, TatB and TatC in complex. Model made using atomic models of individual components as follows: purple—solution NMR data for monomeric *E. coli* TatA, built into a ring and subjected to coarse grain and atomistic molecular dynamics simulation (PDB code 2LZS [102]); yellow—solution NMR structure of truncated (1–101) *E. coli* TatB monomer, with the flexible helices indicated with a yellow arrow (PDB code 2MI2 [101]); cyan—crystal structure of TatC from *Aquifex aeolicus* (PDB code *4B4A* [27]). In each case, the models are aligned with respect to the membrane as per the original study. TatB and TatC have been aligned with respect to each other based on previous cross-linking data [27,104], and using the protein docking algorithm ZDOCK for feasible conformations [105]. Known cross-linking residues are shown as either green (TatB) or purple (TatC) spheres. The putative substrate-binding region is marked with an orange bar. Note that there are currently no known TatBC to TatA oligomer interaction sites.

In contrast to the Sec system, our understanding of protein secretion through the Tat machinery is more limited: how the constituent subunits TatA (with 1 TMH), TatB (1 TMH) and TatC (6 TMH) are capable of delivering a diverse range of large folded proteins across the membrane, without severely compromising the energy-transducing capabilities of the inner membrane, is conceptually difficult to fathom. However, this looks set to change due to a recent turn of events heralding the structures of the core complex TatC [27,28], TatB [101] and the putative protein-translocating subunit TatA [102,103]. The structures of these subunits, arranged according to one possible model for how they come together into an active complex, are shown in figure 7. Note, however, that this model is purely speculative; other models involving alternative arrangements and stoichiometries have also been proposed [106].

Like pre-protein substrates of the Sec system, those of the Tat machinery also contain a cleavable SS [26], recognized in this case by the TatBC sub-complex [107,108]. Also in common with Sec is the utilization of the PMF for secretion [109,110]. Despite this, the transport process is obviously fundamentally different from the passage of unfolded polypeptide through the constricted channel between the two halves of SecY. Moreover, the export process is independent of additional large cytosolic factors, such as an ATP-driven motor: the PMF in this case is necessary and sufficient for transport.

The structure of TatC reveals a glove-like bundle of six TMHs, and provides the basis for our understanding of this process—starting with potential contact sites for TatB and the substrate SS [27,28]. An interesting hypothesis has emerged, whereby the TatBC–pre-protein complex combines with multiple copies of a pore-forming TatA [102] (figures 6*b* and 7). The structure of TatA determined by NMR reveals a short TMH joined to an amphipathic helix by a flexible linker [111]. The propensity of TatA to oligomerization [112] is mediated by the single TMH [102,103] and could potentially form a channel through which proteins cross the membrane. TatBC complexes activated by the pre-protein might promote the nucleation of TatA ring structures. Indeed, molecular dynamics simulations show that both the TatC and oligomers of TatA might bring about a constriction of the membrane [28,102] through which large objects like fully folded proteins could breach. The model proposed by Berks, Schnell and colleagues is attractive because leeway in the oligomeric state of TatA, allowed by the flexibility of the loop and loose association of the amphipathic helix with the membrane surface, could be tailored according to the size of the substrate protein. The constriction of the membrane at the centre of a TatA ring might destabilize a suitably sized membrane patch only transiently. The combination of a snug fitting channel with a transient opening may allow transport without compromising integrity of the membrane. Whether the PMF facilitates the dynamic cycle of the complex assembly and disassembly or acts directly on the substrate itself is unclear.

## 5. Conclusion

Given the maturity of the protein translocation field, its central position in biology and, moreover, the existence for over a decade of the atomic structure of the Sec complex, it is perhaps surprising that the mechanism still eludes us: progress has been slow through these troubled waters. This reflects the fact that protein transport is a complex process involving the passage of a highly variable substrate into and across the membrane, driven both by ATP hydrolysis and the PMF. The structure of the SecYEG–SecA complex bound to an ATP analogue, still the only atomic resolution structure of the translocon with an energy-transducing partner, holds part of the answer. The solution will require the structure of the ADP-bound state, as well as of those associated with a pre-protein. Disentangling the details of this highly dynamic process will likely require many such snapshots at different stages of secretion, and any resulting model must incorporate the functional and kinetic insights discussed above.

Another titanic task for both the Sec and Tat pathways is to elucidate how the PMF can drive translocation—both of unfolded polypeptides and folded domains of widely varying dimensions. Solving this problem will not be plain sailing: many crucial structural and functional assays are incompatible with applying a PMF, and proving a specific proton pathway through a membrane protein is notoriously difficult. Nevertheless, with the new wave of high-resolution EM structures—particularly of translocons caught in the act of secretion—a more complete understanding of the conformational transitions that underpin protein export may be on the horizon.
